# A Novel Core Strengthening Intervention for Improving Trunk Function, Balance and Mobility after Stroke

**DOI:** 10.3390/brainsci12050668

**Published:** 2022-05-20

**Authors:** Rakesh Pilkar, Akhila Veerubhotla, Oluwaseun Ibironke, Naphtaly Ehrenberg

**Affiliations:** 1 Center for Mobility and Rehabilitation Engineering Research, Kessler Foundation, West Orange, NJ 07052, USA; oibironke@kesslerfoundation.org (O.I.); nehrenberg@kesslerfoundation.org (N.E.); 2 ActiGraph, Pensacola, FL 32502, USA; 3 Department of Rehabilitation Medicine, New York University Grossman School of Medicine, New York, NY 10016, USA; akhila.veerubhotla@nyulangone.org

**Keywords:** trunk rehabilitation, core strengthening, hemiplegia, stroke, electromyography, gait and posture

## Abstract

This paper a novel core-strengthening intervention (CSI) delivered using the AllCore360°, a device that targets trunk muscles through a systematic, high-intensity rotating-plank exercise. Three individuals (age: 61.7 ± 3.2 years; range: 58–64 years) with post-stroke hemiplegia participated in 12-sessions of the CSI. The participants completed up to 142 rotating planks at inclination angles (IAs) that ranged from 40° to 65°, over 12 sessions. The interventional effects on the functional outcomes of trunk performance, balance and mobility were assessed using the Trunk Impairment Scale (TIS), the Berg Balance Scale (BBS), the Timed-Up and Go (TUG) test, the 10-m walk test (10MWT), and the 6-min walk test (6MWT). Postural outcomes were assessed using the center of pressure (CoP) data recorded during quiet standing on a balance platform, and neuromuscular outcomes were assessed using electromyography (EMG) during AllCore360° rotations. All participants completed the CSI (minimum of 120 rotations), demonstrating the feasibility of the CSI in chronic stroke. The CoP data suggested improved lateral control of posture during standing across participants (averaging an over 30% reduction in lateral sway), while the EMG data revealed the ability of the CSI to systematically modulate trunk muscle responses. In summary, the current investigation presents the feasibility of a novel delivery method for core strengthening to maximize rehabilitation outcomes in the chronic phase of stroke.

## 1. Introduction

Trunk control is the ability of the trunk muscles to enable the body to maintain an upright posture and to perform weight shifts to maintain balance during static and dynamic postural tasks [1]. Hemiplegia, a secondary consequence of stroke, is characterized by severe unilateral muscle weakness and is linked to compromised mobility, balance, and activities of daily living (ADL), resulting in the loss of independence [2]. Trunk impairments after stroke are multidirectional in hemiplegic patients [1]. These impairments are characterized by weakness and delayed activation of the trunk muscles, significant error in sensing trunk position, inadequate control of the center of pressure (CoP), decreased trunk performance, and trunk kinematic asymmetry during gait [3]. Even in the chronic phase of stroke, weakened activations of trunk flexors and extensors muscles and lower peak torques have been observed compared to healthy individuals [4].

For individuals post stroke, efficient trunk function is essential for postural stability, balance, and the functional mobility needed to perform ADL. Efficient trunk function facilitates proximal stability or control during postural and mobility tasks [5,6], as trunk muscles play a major role in maintaining anti-gravity postures such as sitting and standing, which constitute a major part of ADL. Although leg muscles are partly involved in trunk stabilization in the anterior-posterior (AP) direction, lateral balance and postural stability rely almost exclusively on trunk muscles [7]. Due to the critical role of trunk muscles in maintaining posture, the weakness of trunk muscles post stroke has a significant negative influence on balance and mobility. Evidence suggests that the ability to maintain balance, control posture and walk post stroke depends heavily on trunk function and as such, trunk function is an important functional predictor at hospital discharge [8,9,10].

Trunk function has always been a major point of interest during post-stroke rehabilitation [11,12,13,14], and most current trunk training programs involve core stabilizing, reaching, weight-shifting, or proprioceptive neuromuscular facilitation exercises [6,12,13,14]. These approaches are beneficial for improving static and dynamic balance while sitting, as well as seated arm movements [6,12,13,14] and reducing fall risk [14]. However, many of the postural tasks involved in the aforementioned rehabilitation programs require therapist assistance which could lead to variable support, engagement and inconsistent repeatability, which could, in turn, lead to variability in the dosing of the delivered interventions. As a result, the heterogeneity in dosing and the intervention exercises limits the standardization of treatment and makes it challenging to interpret the interpretation of results across studies [6].

With the recent advancements in rehabilitation technologies, many robotic exoskeletons have been employed in clinical rehabilitation settings [15,16,17,18,19]. The major goal of these devices is to improve walking by facilitating lower extremity motion, thereby increasing step count and walking time (dosing) through intense and repetitive practice. Robotic exoskeletons used for gait and balance training are engaging trunk muscles and are potentially targeting trunk function [19]. However, these secondary impacts on trunk function and neuromuscular output are still under investigation, as information on their effectiveness in trunk training is still lacking [6]. To summarize, most of these devices are predominantly focused on walking, and therefore target the lower limbs to improve neuromuscular and temporal-spatial performance during gait. Such devices seldom specifically target trunk muscles.

There are a few robotic devices that are used for trunk rehabilitation. Min et al. evaluated the therapeutic effects of using a trunk stabilization training robot (3DBT-33) in chronic stroke [20]. Stabilization training was delivered through an instrumented chair and interactive games that triggered weight-shifting and postural coordination movements. The intervention group showed significantly improved functional balance and mobility, supporting the use of trunk-specific rehabilitation training on improving functional outcomes, however, objective outcomes of gait and balance weren’t reported. Another robotic device, *hunova* [21], works on the same principle of trunk rehabilitation, with more degrees of freedom at the base of support (sitting and standing). *Hunova* offers numerous balance and core strengthening programs which have shown to be effective in improving balance and trunk control in chronic stroke [22], although the information on the trunk neuromuscular mechanisms is not clear.

Considering the current limitations of trunk-specific rehabilitation and the major role that the trunk plays in regaining functional independence post stroke, it is important to investigate novel approaches that could provide consistent, task-oriented training that specifically targets trunk muscles and trunk function. The objective of the current investigation is to evaluate the feasibility of a novel, core-strengthening intervention (CSI) program delivered through a novel device, the AllCore360°, specifically designed to target trunk muscles through a task-oriented intense exercise. To our knowledge, this is the first investigation to evaluate the feasibility of this novel intervention in chronic stroke participants in a clinical research setting. The feasibility of the intervention is evaluated using clinical outcomes of static and dynamic balance and mobility. The effect of the intervention on standing is assessed using center of pressure (CoP). We hypothesized that a 4-week core-strengthening program would result in improved clinical outcomes of trunk function, balance, and biomechanical outcome (CoP) in three individuals with post-stroke hemiplegia. As an exploratory analysis, we also aimed to evaluate if this novel core-strengthening paradigm could facilitate modulated responses of trunk muscles based on the changes in the applied intensity of the task. To support this, we assessed electromyography (EMG) data recorded from trunk muscles while all three participants performed on the AllCore360° at predefined settings.

## 2. Materials and Methods

- Case Descriptions

Adults (age: 18–65 years) in the chronic phase of stroke (time since injury > 6 months) were recruited for the study. Three individuals (two males, one female) participated in the study. Inclusion criteria were: (1) must have sustained a stroke at least six months prior to enrollment; (2) no history of injury or pathology for uninvolved limb within the last 90 days; (3) sufficient sitting balance and ability to perform on the AllCore360° device during a practice trial at the screening; (4) be able to walk independently for 10 m without any assistive device; (5) medically stable for three months and with the expectation that current medication can be maintained without drastic change for at least two months; and (6) adequate cognitive function to give informed consent, understand instructions, and provide feedback. Exclusion criteria were: (1) severe cardiac diseases such as myocardial infarction or congestive heart failure; (2) uncontrolled blood pressure; (3) pregnancy (confirmed by pregnancy test); (4) uncontrolled pre-existing history of seizure disorder prior to the most recent episode of stroke; (5) additional orthopedic, neuromuscular, or neurological pathologies that would interfere with the ability to perform the intervention; (6) difficulty following or responding to commands that would limit the study participation; (7) enrolled in another research study or therapy (from a licensed physical therapist) that is likely to affect the outcomes of the current investigation.

The participant characteristics are summarized in Table 1.

### 2.1. Participant S1

A 58-year-old male (weight: 95 kg; height: 170.2 cm; body mass index (BMI): 33) with post-stroke unilateral hemiplegia affecting his left side (time since injury: 18 years) was enrolled. The participant scored 31/56 on the Berg Balance Scale (BBS) at baseline showing poor balance and high fall risk. Particularly, the participant showed impaired balance and postural control in performing sit-to-stand, stand-to-sit, lateral shifts, turns, and single-limb tasks. The Trunk Impairment Scale (TIS) (score: 11 of 23) at baseline also suggested elevated trunk displacements, poor coordination, and postural instability during static and dynamic sitting tasks. However, the participant showed walking speed of 0.8 m/s at the baseline visit, suggesting relatively higher walking function compared to trunk and static balance functions. The participant had high blood pressure which was controlled using medication, but had no history of any other neuromuscular, cardiovascular, or orthopedic injury.

### 2.2. Participant S2

A 64-year-old female (weight: 78 kg; height: 167.6 cm; BMI: 28) with post-stroke unilateral hemiplegia affecting her left side (time since injury: 3.7 years) was enrolled. The participant scored 47/56 on the BBS at baseline showing a lower fall risk. However, balance impairments were observed during sit-to-stand, transfers, and while standing on one foot. The TIS score of 20 showed moderately impaired trunk function and asymmetry during dynamic sitting (rotations) and coordination. Walking speed was measured to 0.88 m/s at baseline, suggesting the participant to be relatively high-functioning post stroke. The participant had high blood pressure which was controlled using a medication, and a prior history of the human immunodeficiency virus (HIV).

### 2.3. Participant S3

A 63-year-old male (weight: 88 kg; height: 182.9 cm; BMI: 27) with post-stroke left hemiplegia (time since injury: 2.7 years) was enrolled. The participant scored 50/56 on the BBS at baseline showing a lower fall risk. Balance impairment and poor postural control were observed while standing on one foot. The TIS score of 17 showed moderately impaired trunk function which was characterized by compensatory movements of the upper extremities during dynamic sitting, as well as asymmetric trunk movements during the coordination tasks. Walking speed measured (0.91 m/s) at baseline suggested a high-functioning individual. In addition, the participant had high blood pressure high cholesterol, diabetes, and peripheral neuropathy (both feet).

B. Core Strengthening Intervention (CSI) program

### 2.4. The Device

**Figure 1:**
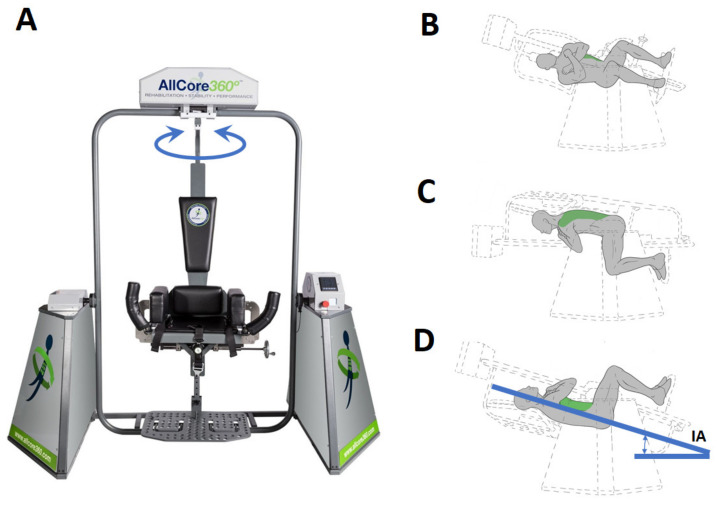
(**A**)The AllCore360° device, and (**B**–**D**) different regions of the core targeted during a single rotation of the AllCore360°. IA—Inclination Angle.

The core-strengthening intervention (CSI) was delivered through the AllCore360° (Figure 1), a commercially available core-training device. The AllCore360° is a device that is specifically designed to facilitate core strengthening through the sustained engagement of trunk muscles during a rotational plank exercise. As shown in Figure 1, the participant sat in the chair which rotated 360° around its central vertical axis. In addition, the outer section of the device enabled the chair to assume a specific posterior inclination angle (IA) from 0–90° while rotating. During a single rotation or ‘spin’, the chair completed a 360° rotation in approximately 80 s, and this angular speed was kept constant for all trials and conditions/IAs. During the spin, participants were asked to hold the body in a plank position without depending on the extremities and without touching their back to the backrest. Figure 1B,C shows the participant positions at different stages of a spin.

### 2.5. Procedure

For all three stroke participants, the IAs for the AllCore360° spins were determined based on the visual observation of participants’ ability to maintain the correct posture (i.e., to hold their posture without touching the backrest). Participants’ feedback on their comfort and safety was also considered. Participants were secured into the chair from the waist down with the body at 90° (i.e., an upright seated position). If needed, the participant was also provided with a trunk strap that was sufficiently loose to allow enough space between the back and the backrest to provide trunk support if needed. All participants were asked to hold their posture (back not touching the backrest) against the gravity while the AllCore360° tilted back to a predefined IA between 0° to 90° and rotated 360°. The spins were performed in either clockwise (CW) or counter-clockwise (CCW) directions. For each participant, each spin was customized (using IA) to present a challenging engaging, and safe training environment. The IA ranges from 90° (the easiest level with the chair fully upright) to 0° (the most difficult level with the back of the chair parallel to the ground, similar to Figure 1D).

At the baseline (pre-intervention) visit, all participants performed three spins at 65° (easy), 55° (moderate) and 45° (difficult) to test the feasibility of performing the spins in a repeated manner. This was repeated at the follow-up (post-intervention) for comparison purpose (see the electromyography (EMG) outcomes). The minimal criteria for the intended dosing were to complete at least 10 rotations (5 CW and 5 CCW) per session, three times a week, for a total of four weeks (12 sessions = 120 spins). All participants successfully completed the 4-week CSI program. The details of each participant’s spin directions, IAs and number of spins are summarized in Table 2. Breaks were given at regular intervals or whenever asked for by the participant, with a single session lasting about 30 min including breaks.

C. Outcome Measures

The following assessments were performed at pre and post CSI visits.

### 2.6. Trunk Impairment Scale (TIS)

The TIS measures the motor impairment of the trunk after a stroke through the evaluation of static and dynamic balance and coordination tasks.

### 2.7. Berg Balance Scale (BBS)

The BBS is a 14-item clinical scale used to evaluate static and dynamic balance. Each item is scored from 0 (lowest level of function) to 4 (highest level of function), with a maximum total score of 56.

### 2.8. Timed up and Go (TUG)

The TUG is a clinical test of functional mobility and is scored as time (seconds) required to complete the mobility task, which consists of going from sitting to standing, walking a short distance to a cone, walking around the cone and returning to the chair and then going standing to sitting.

### 2.9. 10-Meter Walk Test (10MWT)

The 10MWT measures the time needed to walk 10 m at a self-selected safe pace.

### 2.10. 6-Minute Walk Test (6MWT)

The 6MWT evaluates endurance and measures the distance (meters) walked on a flat, hard surface, indoors, in a period of 6 min.

### 2.11. Posturography

The assessment involved collecting CoP data at 100 Hz using a balance platform (Neurocom Equitest Clinical Research System, Natus Medical Inc., Pleasanton, CA, USA) during 120 s of quiet standing (QS). The data were filtered at 4 Hz using a 4th order low-pass Butterworth filter, and the anterior-posterior (AP) and medial-lateral (ML) CoP range and root-mean-square (RMS) were computed.

### 2.12. Electromyography (EMG)

EMG data were collected from selected trunk muscles bilaterally during three CW spins at 45°, 55°, and 65°, at baseline and follow-up. Rectus abdominis (RAB), latissimus dorsi (LD), superior erector spinae (SES) were recorded bilaterally at 1500 Hz using Noraxon DTS wireless EMG system (Noraxon, Scottsdale, AZ, USA). However, left (L)LD EMG data were excluded from analyses due to the channel malfunctioning for one participant. The EMG data were band-pass filtered between 30 to 250 Hz (zero-lag, 4th order Butterworth), and a series of notch-filters (83 Hz and its harmonics) were applied to remove the instrumentation noise observed in the power spectrum. The data were then rectified, and smoothed (3 Hz low-pass filtered). All EMG data are presented in microvolts (µV).

The changes (%) in the clinical and posturography outcomes were calculated as, $100\times \frac{\left(Post-Pre\right)}{Pre}.$

### 2.13. Physical ACtivity Enjoyment Scale (PACES)

PACES is an 18-item scale to assess the enjoyment of physical activity in adults [11]. Scores were determined using a 7-point bipolar rating scale with a maximum possible score of 126. Higher scores reflect a greater level of enjoyment for the training program. PACES was administered at the completion of the CSI.

## 3. Results

### 3.1. Functional Outcomes

Table 3 shows the changes in the outcomes assessing trunk function, balance and mobility after completing 12 sessions of the CSI program. The TIS either increased (+8 points for S1) or remained the same (S2 and S3) for the participants. A change of eight points from baseline to follow up for S1 suggested a clinically meaningful increment of 73% [3,23]. The BBS increased for all three participants, with an increase of 45.2% observed for S1 after the completion of CSI program with a clinically significant change of 14 points [24], and a positive change of two points observed for both S2 and S3. For the TUG, S1’s time improved by 22.7% with a clinically significant change of 5.1 s [25], while S2 showed a small improvement of 4.9% and S3 worsened by 5.3% after the intervention. The 10MWT showed an increased walking speed for S3 (2.5%) while S1 and S2 showed decreased walking speeds (5.3% and 11.6%, respectively) post intervention. S2 and S3 showed small distance improvements (1.5% and 2.6%, respectively) in the 6MWT, while S1′s distance decreased by 4.5%.

### 3.2. Posturography Outcomes

**Figure 2:**
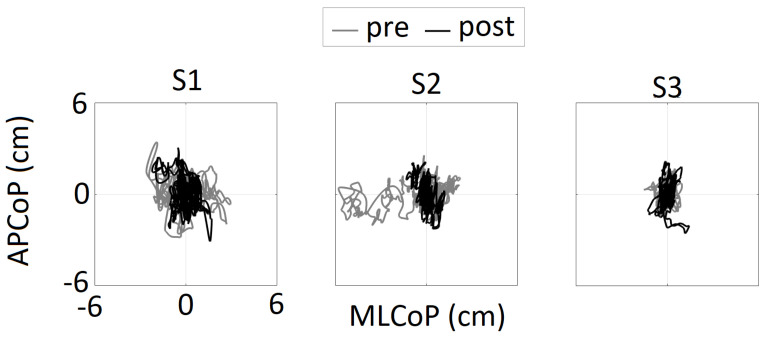
Statokinesiogram representations of all three participants showing the center of pressure (CoP) excursions in anterior-posterior (AP) and medial-lateral (ML) directions. APCoP—anterior-posterior center of pressure; MLCoP—medial-lateral center of pressure.

Biomechanical changes were assessed using the QS CoP data from before and after the CSI. As shown in Figure 2, the statokinesiograms (x axis: medial-lateral (ML) CoP, y axis: APCoP) for all three participants showed unstable postural control characterized by large variability and excursions, which were particularly apparent in the ML direction. Post-intervention data showed reduced CoP excursions in the ML direction for all participant, which were quantified in terms of %change and are presented in Table 4. For S1 and S2, the MLCoP range decreased by 29% and 69%, respectively, and the MLCoP RMS decreased by 32% and 72%, respectively.

### 3.3. Neuromuscular Outcomes

**Figure 3:**
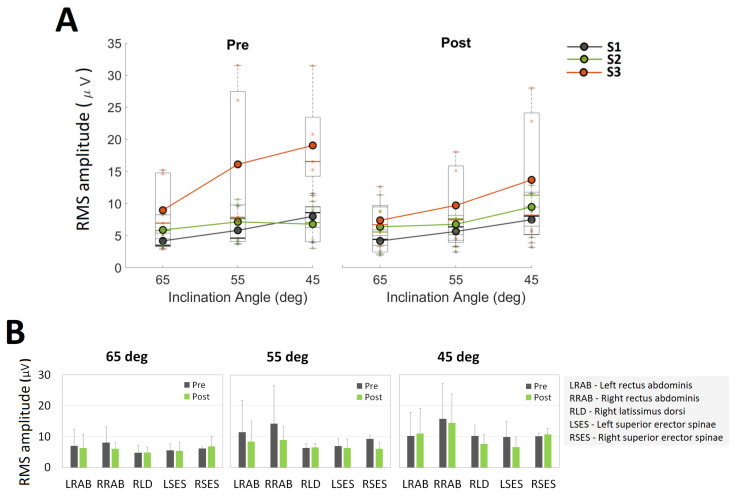
(**A**) Box plot representation of electromyography (EMG) data for all three participants. EMG data from all channels are consolidated into a single dataset for each IA for every participant, (**B**) the mean EMG root-mean-squared (RMS) amplitude for all participants for each muscle group at each IA, before and after the intervention. Error bars represent the standard deviation.

Figure 3A shows the box plot representation of EMG data for each participant. For each participant, EMG data from all channels were combined for each IA. In general, it was observed that the inclination angle of 45° (highest difficulty) elicited higher levels of activations compared to the IA of 65° (lowest difficulty). This relationship between the IA and the levels of elicited muscle contractions was mostly consistent for S1 and S3 at baseline and for all participants at the follow up visit. Figure 3B shows the mean EMG RMS levels for all participants pre and post CSI. It was observed that the neuromuscular responses varied across participants, particularly for RAB muscles. Although several muscle groups showed increased activations (RSES at 65°, RLD at 55°, and LRAB and RSES at 45°), no definite conclusions can be drawn as no maximum volitional contractions (MVC) normalizations were performed.

### 3.4. Physical Activity Enjoyment Scale (PACES)

Scores of 71, 60 and 60 (for S1, S2 and S3, respectively) on the PACES administered at the completion of the intervention showed high levels of satisfaction and enjoyment from participation in the CSI program.

## 4. Discussion

The objective of the current investigation was to evaluate the feasibility of the CSI program delivered through the AllCore360°, a novel core-strengthening device designed to target trunk muscles through a task-oriented and high-intensity exercise program. The current investigation makes significant contributions to the following three aspects of trunk rehabilitation post stroke: (1) the novelty and specificity of the CSI program (and the delivery medium) to target trunk muscles; (2) the feasibility of the CSI in chronic stroke population; and (3) a diverse set of clinical, biomechanical and neuromuscular outcomes and resultant changes therein suggesting the potential of the CSI program to modulate functional and biomechanical mechanisms in a chronic stroke population.

The novel design of AllCore360° enables the presentation of various difficulty levels in terms of IAs. These IAs can be set based on the patient’s functional status, comfort and safety, making the intervention truly patient-specific. The IAs for all participants in this investigation ranged from 65° (easy) to 35° (difficult). Only S2 was able to perform spins at 35° (Table 2), however, IAs between 40° to 65° still provided a safe yet challenging training environment. Further, these difficulty levels directly modulated the neuromuscular responses (Figure 3A) and hence can be used to maximize the engagement of the relevant trunk muscles and neuromuscular outputs. It has been suggested that insufficient recruitment of high-threshold motor units at high angular velocities and disuse of muscles can contribute to trunk impairment [4], and the CSI program presents a unique environment to not only engage trunk muscles that may be underutilized but also enhance their activations using gravitational forces in a non-impact, symmetrical, and coordinated fashion. Such an opportunity is highly significant for facilitating neuroplasticity and pushing the boundaries of functional recovery even in the chronic phase of stroke. Ballester et al. have suggested that there is the possibility of enhanced sensitivity to treatment that extended beyond 12 months post stroke [27]. Therefore, highly-engaging and stimulating intervention can be very significant for individuals with chronic stroke to maintain physical function and quality of life. The consistency of the CSI program is characterized by the resistance provided, which comes from the patient’s own weight due to gravity as opposed to inconsistent therapist-provided resistance, which is the case in many core-strengthening exercise-based interventions [3,13,14,23]. The electromechanical design of the device facilitates repeatability and consistent dosing for optimal outcomes.

The changes observed in some of the functional outcomes post intervention were promising and clinically meaningful. The changes observed for S1 were the most promising (+8 for TIS, +14 for BBS, 22.7% for TUG) and clinically meaningful considering the previously reported post-intervention changes in stroke populations [3,24,25]. S1 showed greater improvement post intervention compared to S2 and S3, though this may have been since the functional statuses of S2 and S3 were higher than S1 (see TIS, BBS and TUG scores in Table 3) at the start of the study. While the results of the current case-series investigation are promising for some participants, these results need to be interpreted cautiously due to the small sample size.

Increased postural sway and asymmetry during standing have been associated with poor balance in individuals with stroke [28]. Marigold et al. found that greater asymmetry was moderately related to increased ML sway in individuals with stroke [29]. We found that all three participants showed reduced CoP excursions in the ML direction post CSI. The intervention did not specifically target the lower limb mechanisms. Therefore, the reduced postural sway in the ML direction could have resulted from better control of the trunk in the ML direction, as lateral postural stability relies almost exclusively on trunk muscles [7].

EMG reflects the status of neuromuscular control of the central and peripheral nervous systems, and as a result, any abnormality in EMG may suggest deficits in motor control in stroke patients [30]. The EMG results were the variable across participants and muscle groups, with the most significant observation being the ability of the CSI to modulate neuromuscular response as a function of IA. The variability in the data and the absence of EMG normalization using MVC levels significantly restrict the pre-post comparison of muscle activations and the presence or absence of neuromuscular adaptations resulting due to the CSI.

### Limitations

One of the major limitations of the current study is the small sample size. Being a case series, the results are not accompanied by statistical analyses and the pre-post differences are reported only in terms of %change. In the future, a large-sample design should be implemented with an age-matched control group to evaluate the effectiveness of the CSI program. The authors acknowledge the absence of hemodynamic outcomes in this case series. Previously, Palevo et al. have reported metabolic outcomes (oxygen consumption (VO_2_), carbon dioxide volume, respiratory quotient), heart rate, blood pressure, and rate of perceived exertion in healthy adults with AllCore360° [31]. It is important to assess these physiological changes in stroke populations undergoing an exercise program such as CSI. Although we monitored the heart rate and blood pressure at regular intervals for patient safety purpose, these data were not quantified. Lastly, all participants were moderate to high functioning individuals and community ambulators (gait speed 0.76–0.94 m/s). Therefore, the feasibility of using this device for individuals in the acute phase of stroke with severe motor impairments is not clear.

Nonetheless, previous research has shown that core-strengthening has the potential to improve trunk function, balance, and functional mobility in individuals post stroke. Currently, there is a large amount of heterogeneity (in terms of types of exercises, intensity and duration of training) across studies that evaluate trunk training protocols [6]. The current investigation presents the feasibility of a novel delivery method which can provide potentially homogeneous, task-oriented and engaging training which could maximize stroke rehabilitation outcomes even in the chronic phase.

## Figures and Tables

**Table 1:** AllCore360° rotations. TSI, time since injury; BMI, body mass index.

ID	TSI (Years)	Age (Years)	Sex	Height (cm)	Weight (kg)	BMI
S1	18	58	Male	170.2	95	33
S2	3.7	64	Female	167.6	78	28
S3	2.7	63	Male	182.9	88	27

**Table 2:** AllCore360 rotations (spins) performed over 12 sessions of intervention. CW, clockwise; CCW, counter-clockwise.

Participant	Direction	Inclination Angle (Deg)							Total
		65	60	55	50	45	40	35	
**S1**	CW	1	0	20	0	34	16	0	71
	CCW	1	0	20	0	34	16	0	71
	**Total**	**2**	**0**	**40**	**0**	**68**	**32**	**0**	**142**
**S2**	CW	11	3	16	3	17	9	1	60
	CCW	11	3	16	3	17	9	1	60
	**Total**	**22**	**6**	**32**	**6**	**34**	**18**	**2**	**120**
**S3**	CW	10	3	16	6	21	4	0	60
	CCW	10	3	16	6	21	4	0	60
	**Total**	**20**	**6**	**32**	**12**	**42**	**8**	**0**	**120**

Bold and highlighted rows represent the total number of spins performed at each IA for each participant. Highlighted column represents the total number of spins performed in each direction. The intersection of highlighted rows and columns represents the total spins (in bold) during the entire intervention period.

**Table 3:** Changes in functional clinical outcomes for three stroke participants after the completion of four-week CSI (core-strengthening intervention) program.

Assessments	Participant	Baseline	Follow Up	Change (Difference, %Change)	Reference Values for Meaningful Changes
Trunk Impairment Scale	S1	11	19	(8, 72.7%)	
	S2	20	20	0	4 ^a^
	S3	17	17	0	
Berg Balance Scale	S1	31	45	(14, 45.2%)	
	S2	47	49	(2, 4.3%)	2.5 to 4.6 ^b^
	S3	50	52	(2, 4%)	
Timed-Up and Go (s)	S1	22.6	17.5	(−5.1, −22.7)	
	S2	13.6	14.3	(0.7, 5.2%)	2.9 ^c^
	S3	12.7	12.1	(−0.6, −4.9)	
10-m walk test (m/s)	S1	0.8	0.76	(−0.04, −5.3%)	
	S2	0.88	0.78	(−0.1, −11.6%)	0.05 to 0.1 ^d^
	S3	0.91	0.94	(0.04, 2.5%)	
6-min walk test (m)	S1	251.8	240.4	(−11.4, −4.5%)	
	S2	287.5	291.9	(4.4, 1.5%)	20 m to 50 m ^e^
	S3	250.8	257.2	(6.4, 2.6%)	

^a^ Significant difference reported by [3,23]. ^b^ Significant difference reported by [24]. ^c^ Significant difference reported by [25]. ^d^ Significant difference reported by [26]. ^e^ Minimal Clinical Important Difference (MCID) reported by [26].

**Table 4:** Changes in CoP outcomes for three stroke participants after the completion of 4-week core strengthening intervention; sd, standard deviation. APCoP—anterior-posterior center of pressure, RMS—root-mean-squared, MLCoP—medial-lateral center of pressure.

	% Change			
	APCoP Range	APCoP RMS	MLCoP Range	MLCoP RMS
S01	−1.49	10.98	−29.24	−32.30
S02	−5.18	17.83	−68.55	−72.10
S03	52.12	26.23	7.63	−1.75
mean	15.15	18.35	−30.05	−35.38
sd	32.07	7.63	38.10	35.28

## Data Availability

The data reported in this investigation can be made available upon request in accordance with Kessler Foundation policies and data sharing agreement.
